# Economic burden of antimicrobial resistance on patients in Pakistan

**DOI:** 10.3389/fpubh.2025.1481212

**Published:** 2025-02-25

**Authors:** Numrah Safdar, Saad Saleem, Muhammad Salman, Ahmad Hussen Tareq, Sidra Ishaq, Saadia Ambreen, Abdul Hameed, Muhammad Bilal Habib, Tariq Mahmood Ali

**Affiliations:** ^1^National Institute of Health, Islamabad, Pakistan; ^2^Federal Government Polyclinic Hospital, Islamabad, Pakistan; ^3^Health Services Academy, Islamabad, Pakistan

**Keywords:** antimicrobial resistance, economic burden, bloodstream infections, admitted patients, Pakistan

## Abstract

**Background:**

Antimicrobial resistance (AMR) occurs when microbes undergo changes that render antimicrobial drugs ineffective against them, resulting in limited, more expensive treatment options, longer hospital stays, and increased mortality rates. No study has estimated the costs related to AMR in hospitals in Pakistan. This study was conducted to determine the financial burden of antimicrobial resistance (AMR) in Pakistan and to compare it with the additional costs incurred by patients who respond well to antimicrobial treatments. The study also aimed to identify the most frequent types of microbes that cause bloodstream infections in Pakistan.

**Methods:**

This quantitative study was conducted employing a prospective cohort study design. A sample of 193 patients was selected from two public sector tertiary care hospitals in the twin cities of Islamabad and Rawalpindi for a period of 7 months. The frequency trends of antimicrobial resistance against 12 blood pathogens were determined by analyzing culture sensitivity reports from patients who tested positive, as provided by pathology laboratories of the study hospitals. Direct and indirect costs were calculated using data from patients’ medical records and through direct interactions with patients.

**Results:**

This study estimated that treating cases of AMR resulted in approximately USD 33.97 [Pakistani Rupees (PKR) 9483.2] in additional costs compared to treating susceptible infections due to extended lengths of hospital stays. However, indirect costs such as spending on food, productivity loss, and accommodation are USD 55.84 (PKR 15588.3) higher in the non-infected control cohort compared to the cases. Direct costs (transport, pharmacy, and laboratory expenses) are directly related to AMR and add an additional burden of USD 12.30 (PKR 3435) for cases compared to non-infected controls. In comparison to susceptible controls, cases incur an additional cost of USD 32.9 (PKR 9185.9).

**Conclusion:**

This study helped predict the economic burden of antimicrobial resistance in admitted patients with bloodstream infections (BSIs) in low- and middle-income countries, such as Pakistan, by different variable cost estimates. These findings will help in designing the most appropriate approach to combat AMR. Additionally, this study serves as a baseline tool that can be extrapolated to estimate the national economic burden because of AMR.

## Introduction

AMR refers to the inherent ability of microorganisms to develop resistance to medications that are designed to impede their growth (1). The global health community recognizes antimicrobial resistance (AMR) as a significant and urgent threat to all forms of life, including humans, animals, and the environment. Previous studies have attempted to quantify the impact of AMR on various factors, including the severity of infections, mortality rates, length of hospital stays, and healthcare costs. However, these analyses have typically focused on specific pathogens and their associated drug combinations in particular regions (2). The threats posed by AMR are extending beyond developing countries, leading to the failure and inability to treat infectious diseases effectively with antibiotics. This situation creates an uncertain future for healthcare systems (3). Infections caused by resistant organisms result in compromised health and poorer outcomes as compared to infections caused by susceptible organisms. In addition to the costs incurred by patients, antimicrobial resistance also imposes health and economic burden on their caretakers, such as out-of-pocket expenses, travel costs, accommodation and food expenses, loss of wages and leisure time, increased anxiety and depression, fear of infections, stigmatization due to limited treatment options, and side effects from last-line antimicrobials (4).

Multiple studies indicate that the most common types of adverse events affecting hospitalized patients include adverse drug events, healthcare-associated infections (HCAIs), and surgical complications (5). Out of every 100 hospitalized patients, seven patients in developed countries and 10 patients in emerging countries acquire an HCAI (6). Hospital-acquired infections (HAIs) are frequent adverse consequences of healthcare systems, threatening the health of both patients and healthcare workers (HCWs), but they can be prevented. According to the Centers for Disease Control and Prevention (CDC) in the USA, common types of HAIs include ventilator-associated pneumonia, central line-associated bloodstream infections, catheter-associated urinary tract infections, surgical site infections (SSIs), and *Clostridioides difficile.* The US CDC estimated that nearly 1.7 million patients acquire healthcare-associated infections annually while being treated for other health issues during their hospital stays, and more than 98,000 patients (1 in 17) die due to these HAIs (CDC). The annual costs for HCAIs alone in the USA are between USD 28 billion and USD 45 billion. Despite this significant expenditure, 90,000 lives are still lost per year, making HCAIs one of the top five causes of death in the USA (5).

According to a UK report, it is estimated that AMR will result in an additional 10 million deaths globally each year by 2050 (7). Another CDC report highlighted that over two million individuals in the United States fall ill due to antibiotic-resistant diseases each year, leading to a minimum of 23,000 deaths (8). AMR is progressively becoming a burden on healthcare systems and society as a whole, and published estimations of its clinical and economic impact exhibit significant variations (9). The World Bank released a report in 2017 highlighting that the annual GDP could decline by 1.1% by 2050, with an annual shortfall of USD 1 trillion by 2030. In the worst-case scenario, the annual GDP could decline by 3.8% by 2050, with an annual shortfall of USD 3.4 trillion by 2030 (10). It is believed that at a macroeconomic level, reduced productivity due to illness or death among working populations can result in the loss of gross domestic product (GDP) (11). Another study reported a decline in public trust in the health system due to the impact of AMR on society (12).

Pakistan is significantly lacking in legislation and policies to optimize the use of antimicrobials (National Action Plan on AMR-2017). The National Action Plan on Antimicrobial Resistance has highlighted various significant factors that contribute to AMR. These factors include unnecessary registered products (approximately 50,000), misleading advertisements (with only approximately 15% of promotional brochures meeting the WHO criteria), over 50% self-medication in Pakistan’s population, and a higher prevalence of quackery in the country. Prescriptions often include more than three drugs per patient (a practice known as polypharmacy), including 70% of antibiotics prescribed by authorized physicians. Additionally, over-the-counter (OTC) availability of antibiotics is very common, which poses a significant challenge when using potent antibiotics for highly resistant infections (13). A situation analysis report on antimicrobial resistance in Pakistan, introduced by the Pakistan Global Antibiotic Resistance Partnership, highlights similar challenges and concerns, in addition to irrational prescription practices among physicians, who often favor expensive broad-spectrum antibiotics, inadequate surveillance systems which lack the necessary experts, and the uncontrolled use of antibiotics in poultry, animals, and agriculture (13).

In line with WHO recommendations and SDG 3.d.2 indicators, bloodstream infections are largely disseminated and frequently observed in healthcare facilities. A point prevalence study on HAI burden from Pakistan suggested that the most frequent infections found in admitted patients are bloodstream infections (BSIs), followed by pneumonia, ear, eyes, nose, and throat infections, and skin and soft tissue infections (14). Bloodstream infections exhibit the most standardized testing methodology as compared to other HAIs. In this study, we selected BSI with the objective of assessing the financial burden on patients associated with antimicrobial resistance. We measured the difference in treatment costs between patients with resistant and susceptible isolates and assessed the trends of antibiotic resistance within the context of Pakistan. This study reflects a pilot effort to assess the economic consequences of antimicrobial resistance, as no similar study has been conducted in the country until now.

AMR is on the rise globally and has significant financial implications. The additional costs related to AMR have been estimated in some countries. This pioneering study in Pakistan will help policymakers understand the additional yet preventable AMR burden on the general public and the country as a whole through simple interventions. The implementation of the National Action Plan on Antimicrobial Resistance in Pakistan plays a vital role in addressing this emerging threat and the burden caused by AMR.

## Methodology

This study was quantitative in nature. A prospective cohort design was adopted to estimate the study objectives. The study was conducted over a 7-month period, from November 2022 to June 2023, at the two most crowded public-sector tertiary care hospitals in the twin cities of Pakistan (Hospital A and Hospital B). Data were collected over a period of 4 months, from January 2023 to April 2023, after obtaining ethical approval from the respective committees.

### Ethical approval and patients’ consent

Ethical approval for the research study was obtained from the Institutional Review Board of the Health Services Academy, after receiving administrative approval from the relevant institutional heads.

Informed formal consent was obtained from all respondents (patients) before enrolling them in the study. The objectives and study plan were explained to them, and the anonymity and confidentiality of the patients were ensured.

### Inclusion and exclusion criteria

Admitted patients who were tested for blood culture sensitivity (c/s) in the hospital were included in the study, regardless of the positive or negative results. Patients whose total length of stay (LOS) in the hospital and death were confirmed during the study period were included. Costs on patient’s end was calculated only. Patients with infections other than bloodstream infections were excluded from this study. Costs related to AMR at the hospital and for patients who died after being discharged from the hospital were not considered.

**Figure 1:**
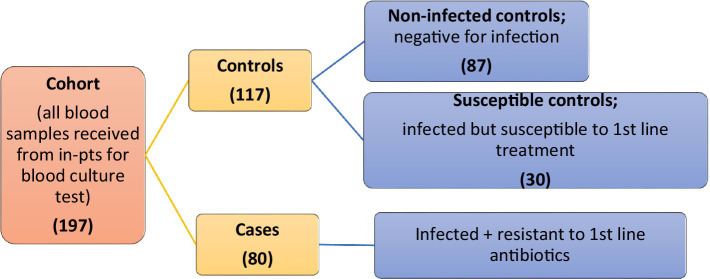
Categorization of sample population.

A simple non-probability sampling technique was used. A total of 197 cohorts was collected from both hospitals. The initial cohort was divided into 80 cases and 117 control groups. Cases were considered infected and resistant to first-line antibiotics. Controls were further classified into two groups: non-infected controls (*n* = 87), who were negative for infection, and susceptible controls (*n* = 30), who were infected but responsive to first-line treatment (Figure 1).

Data were collected using a questionnaire that included all the variables required to meet the study objectives. The questionnaire was designed using the KOBO tool developed by the WHO and was face-validated by seeking expert opinions. The questionnaire contained generic information such as demographics (name, PCN number, age, sex, ward, area of residence, occupation, and social status as income) and cost-specific parameters in terms of direct costs (treatment cost, diagnostic cost, and transportation cost) and indirect costs (food expenses, accommodation costs, and productivity loss) for admitted patients. The required information was gathered through patient medical records (PMR) and interactions with patients. This study included all bacterial pathogens responsible for bloodstream infections in these two public sector hospitals. These 12 pathogens were categorized into 10 codes, depending on their occurrence in the sample. The 10th code included three rare bacteria.

Among the direct costs, the transportation cost included fuel charges for personal vehicles, charges for rental cars, taxi fares, and ambulance charges, particularly in cases involving serious patients and fatalities. Treatment costs comprised the cost of all antibacterial drugs administered to the patient, and diagnostic costs included charges of blood culture and antibiotic susceptibility tests. Indirect costs included all expenses related to meals and stays outside the hospital for caretakers or family members traveling from distant location to visit admitted patients, as public sector hospitals provide free meals for the admitted patients only.

The data were then cleaned and analyzed using IBM Statistical Package for the Social Sciences (SPSS)-21 and Strata for both descriptive and inferential statistics. For observing the impact of antimicrobial resistance on admitted patients, variables such as length of stay (LOS), mean staff time used, and mortality frequency were also used. To measure the difference in means for AMR-attributed variables between the two study cohorts, that is, case cohort and control cohort (susceptible and non-infectious controls), a *t*-test was performed at a 95% confidence interval. A *p* < 0.05 was considered statistically significant. Statistically significant results were denoted by asterisks.

## Results

### Descriptive statistics

A major part of the sample included those in the age group of 16–25 years. Out of the total population of 197 from both hospitals, 97 (49.2%) were women, and the remaining 100 were men, accounting for 50.8% of the sample. Among the total sample, 45% were non-infectious controls with no growth of bacteria, and 15% were susceptible controls. A total of 80 individuals (41%) among 197 were the cases. We observed that the female population was more resistant to antibiotics. Moreover, the trend of antimicrobial resistance among the wards indicates that medical wards have the highest occurrence of resistant profiles, while critical care units (CCU) exhibit the lowest, as shown in Table 1.

**Table 1:** Distribution of AMR among the sample.

Variable	Category	Cases	Controls		Total *N* %
		AMR Cohort *N* %	Non-infectious cohort *N* %	Susceptible cohort *N* %	
Treatment ward	1:MU	41 (42.71)	36 (37.50)	19 (19.79)	96 (48.7)
	2: SU	5 (31.25)	4 (25.00)	7 (43.75)	16 (8.1)
	3: ER	9 (90.00)	1 (10.00)	0 (0.00)	10 (5.1)
	4: CCU	1 (12.50)	5 (62.50)	2 (25.00)	8 (4.1)
	5: PICU, SICU, NICU, MICU	18 (41.86)	24 (55.81)	1 (2.33)	43 (21.8)
	6: PHDU, PEADS, MCH, MOTHERROOM	6 (25.00)	18 (75.00)	0 (0.00)	24 (12.2)
OPD visits	No	30 (48.39)	25 (40.32)	7 (11.29)	62 (31.5)
	1	49 (37.69)	61 (46.92)	20 (15.38)	130 (66)
	2	1 (20.00)	2 (40.00)	2 (40.00)	5 (2.5)
Health insurance	No	67 (42.41)	65 (41.14)	26 (16.46)	158 (80.2)
	Yes	13 (33.33)	23 (58.97)	3 (7.69)	39 (19.8)
Mortality	No	59 (39.07)	70 (46.36)	22 (14.57)	151 (76.6)
	Yes	21 (45.65)	18 (39.13)	7 (15.22)	46 (23.4)
	Total	80 (40.61)	88 (44.67)	29 (14.72)	197 (100)

**Figure 2:**
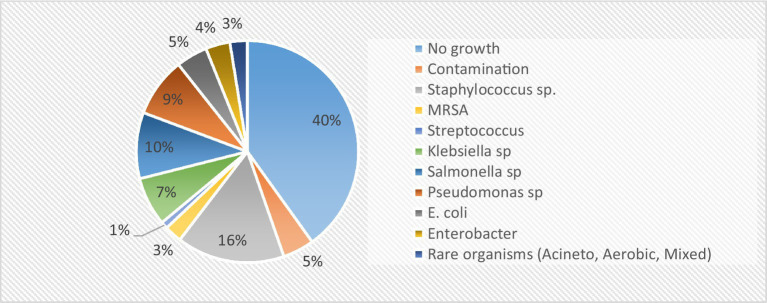
Pathogen distribution in bloodstream infections.

Furthermore, the majority of the admitted patients had the highest percentage of single outpatient department (OPD) visits (66%). Approximately 80.2% of the admitted patients were not health-insured, and those with insurance utilized the Sehat Sahulat scheme. Mortality, measured as the proportion of patients who died during the study, was the highest in the AMR cohort (45.65%). As shown in Table 2, the most frequent blood pathogen was *Salmonella* sp., followed by *Pseudomonas* sp., *Klebsiella* sp., *Escherichia* sp., *Enterobacter* sp., and *MRSA* species. The frequency of pathogens is shown in Figure 2. *Staphylococcus* spp. was the most common pathogen among the susceptible cohorts.

**Table 2:** Frequency of bacterial blood pathogens across both hospitals.

Variable	Category	Cases	Control		Total *N* %
		AMR cohort *N* %	Non-infectious cohort *N* %	Susceptible cohort *N* %	
Bacterial isolate	0: No growth	0 (0.00)	79 (100.00)	0 (0.00)	79 (40.1)
	1: Contamination	0 (0.00)	9 (100.00)	0 (0.00)	9 (4.6)
	2: *Staphylococcus* sp.	2 (6.45)	0 (0.00)	29 (93.55)	31 (15.7)
	3. MRSA	5 (100.00)	0 (0.00)	0 (0.00)	5 (2.5)
	4: *Streptococcus*	2 (100.00)	0 (0.00)	0 (0.00)	2 (1.0)
	5: *Klebsiella* sp.	14 (100.0)	0 (0.00)	0 (0.00)	14 (7.2)
	6: *Salmonella* sp.	19 (100.0)	0 (0.00)	0 (0.00)	19 (9.6)
	7: *Pseudomonas* sp.	17 (100.0)	0 (0.00)	0 (0.00)	17 (8.6)
	8: *E. coli*	9 (100.00)	0 (0.00)	0 (0.00)	9 (4.6)
	9: *Enterobacter*	7 (100.00)	0 (0.00)	0 (0.00)	7 (3.6)
	10: Rare organisms (Acineto, Aerobic, Mixed)	5 (100.00)	0 (0.00)	0 (0.00)	5 (2.5)
	Total	80 (40.61)	88 (44.67)	29 (14.72)	197 (100.0)

## Inferential statistics

In the assessment of other consequences related to AMR, it was found that the mean length of stay (LOS) for the AMR cohort was 7.9 days (95% CI: 6.4–9.5), which is shorter than that of the non-infectious cohort (11.1 days, 95% CI: 8.4–14.1), with a significant difference of −3.2 days (*p < 0.05). The LOS after CST report for the AMR cohort was 5.15 days (95% CI: 3.8–6.5), which was significantly higher than that in the non-infectious cohort (0.068 days, 95% CI: −0.1–0.2) (**p < 0.05). The mean staff time used was 687.3 min for the AMR cohort and 693.1 min for the non-infectious cohort, with a non-significant difference of −5.8 min (*p* > 0.05). The mean number of laboratory tests for the AMR cohort was significantly higher than both the non-infectious and susceptible cohorts (**p < 0.05). The mean number of OPD visits showed no significant difference between the AMR and non-infectious cohorts but was significantly lower in the AMR cohort than in the susceptible cohort (**p < 0.05), as depicted in Table 3.

**Table 3:** Comparison of other AMR consequences among cases and controls.

	AMR cohort cases (*n* = 80) 95% CI	Non-infectious cohort (*n* = 88) 95% CI	Difference (Case-susceptible) 95% CI	Susceptible cohort (*n* = 9) 95% CI	Difference (case-susceptible) 95% CI
Mean LOS in days (overall)	7.9 (6.4–9.5)	11.1 (8.4–14.1)	−3.2* (−6.6–0.05)	7.2 (5.6–8.8)	0.7 (−2.0–3.5)
Mean LOS in days (after positive C/S report)	5.15 (3.8–6.5)	0.068 (−0.1–0.2)	5.1** (3.8–6.4)	5.1 (3.6–6.5)	0.08 (−2.4–2.5)
Mean staff time used in minutes	687.3 (410.0–964.4)	693.1 (485.0–901.1)	−5.8 (−346–334.3)	342 (254.5429.6)	345** (−117.5–807.8)
Mean no. of laboratory tests	1.98 (1.9–2.0)	1.056 (1.0–1.1)	0.93** (0.9–1.0)	1.0 (1.0–1.1)	1.0** (0.9–1.0)
Mean no. of OPD visits	0.63 (0.5–0.8)	0.73 (0.6–0.8)	−0.10 (−0.3–0.05)	0.8 (0.6–1.0)	−0.2** (−0.41–0.03)

(*) shows 1% level of significance; (**) for 5% level of significance; (***) for 10% level of significance.

**Figure 3:**
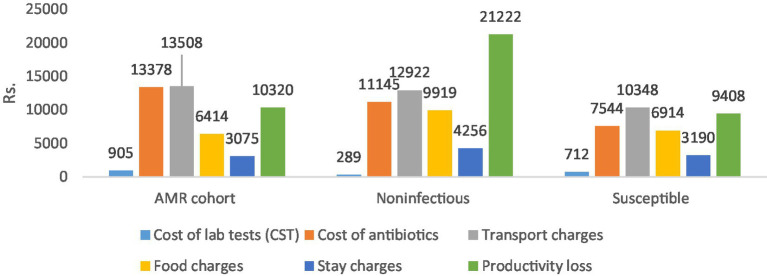
Cost comparison between all three cohorts.

Table 4 shows the differences in the direct and indirect costs across the case and control cohorts. The costs of laboratory tests (diagnostics), antibiotics (therapeutics), and transportation charges were significantly higher for the AMR cohort compared to both control cohorts (**p < 0.05), as shown in Figure 3 below.

**Table 4:** Cost comparison between cases and controls.

Variable		AMR cohort (*n* = 82) 95% CI	Non-infectious cohort (*n* = 88) 95% CI	Difference (case-non-infectious) 95% CI	Susceptible cohort (*n* = 9) 95% CI	Difference (case-susceptible) 95% CI
Direct Cost	Cost of lab tests (CST)	905 (858.1–951.8)	289 (230.3–346.9)	616** (541.1–691.6)	712 (687.3–736.8)	193** (113.8821–272)
	Cost of antibiotics	13,378 (6295.9–20459.3)	11,145 (8278.201–14011.8)	2,233 (−5099.1–9564.4)	7,544 (4819.4–10268.5)	5,833 (−6020.6–17,688)
	Transport charges	13,508 (10178.5–16836.5)	12,922 (10512.6–15330.6)	586 (−3440.1–4611.9)	10,348 (7947–12749.5)	3,159 (−2535.5–8,854)
	Subtotal	27,790 (18602.15–36978.1)	24,355 (20038.82–8671.6)	3,435 (−6347.53–13217.4)	18,604 (14552.4–22656.1)	9185.9 (−6236.7–24608.4)
Indirect Cost	Food charges	6,414 (4588.3–8239.2)	9,919 (6133.1–13705.6)	−3505** (−7812.2–801.1)	6,914 (4902.3–8925.3)	−500 (−3744.1–2,744)
	Stay charges	3,075 (1613.4–4536.6)	4,256 (2111.8–6399.6)	−1,181 (−3807.7–1446.3)	3,190 (997.2–5382.1)	−115 (−2850.5–2621.2)
	Productivity loss	10,320 (7552.437–13087.8)	21,222 (9066.608–33377.7)	−10902** (−23836.84–2032.7)	9,408 (6123.9–12692.1)	912 (−4056.5–5880.6)
	Subtotal	19,809 (14588.1–25029.6)	35,397 (18616.6–52177.8)	−15588.3** (−33758.7–2582.0)	19,512 (13786.9–25236.1)	297.4 (−8974.6–9569.3)
Total	(Direct cost + Indirect cost)	47,599 (34156.4–61041.6)	59,752 (39981.3–79523.4)	−12153.4 (−36361.6–12054.8)	38,116 (29698.7–46532.8)	9483.2 (−13343.7–32310.1)

(*) shows 1% level of significance; (**) for 5% level of significance; (***) for 10% level of significance.

**Figure 4:**
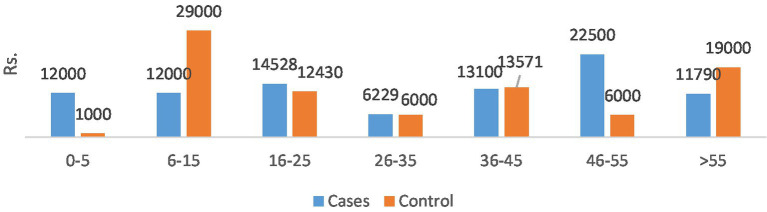
Age group wise productivity loss.

The total direct costs were significantly greater for the AMR cohort compared to the non-infectious cohort. The indirect cost comparison showed that food charges were significantly lower in the AMR cohort compared to the non-infectious cohort (**p < 0.05). The productivity loss was significantly greater in the non-infectious cohort compared to the AMR cohort (**p < 0.05). Age-wise productivity losses for both cases and controls are shown in Figure 4. The indirect subtotal cost was significantly higher for the non-infectious cohort compared to the AMR cohort. The total medical cost, including both direct and indirect costs, was the highest for the non-infectious cohort, indicating a prolonged stay in hospitals.

Moreover, Table 5 shows the comparison of AMR-related consequences across both hospitals (A and B). There was no significant difference in the mean direct cost, mean total cost, mean productivity loss, or mean staff time between the two hospitals. However, the mean indirect cost was significantly higher for Hospital B compared to Hospital A (**p < 0.05).

**Table 5:** Comparison of AMR-related consequences across both hospitals.

Hospitals	Mean direct cost	Mean indirect cost	Mean total cost	Mean productivity loss	Mean staff time used
HFH	25363.02	14314.89	39677.92	19188.52	712.1809
PIMS	24484.16	9261.165	33745.33	11284.2	572.2816
Mean difference	878.86	5053.725	5932.59	7904.325	139.8993

Median values, along with intraquartile ranges, were calculated for total cost, direct cost, indirect cost, and productivity loss, mentioned in the sequence: median values as PKR 2808, 19,426, 6,000, 7,100 and IQR values as PKR 38471, 20,750, 13,000, 11,300.

Further details related to the distribution of transport costs, food charges, stay charges, and total productivity loss across age, sex, hospitals, treatment wards, health insurance coverage, types of bacteria detected, laboratories performed, number of OPD visits, and mortality frequency among case and control cohorts were recorded and can be provided upon request from the author.

## Discussion

The incidence and prevalence of bacterial diseases resistant to antibiotics have reached unprecedented heights in the 21st century, posing a latent pandemic threat to global public health and demanding immediate action. Anybody, regardless of age or gender, can become resistant to antibiotics in any nation. The WHO has recognized AMR as one of the top three greatest dangers to public health. After cardiovascular disorders, antimicrobial-resistant infections are the third most common cause of death. Methicillin-resistant *Staphylococcus aureus* (MRSA) is a well-known example of the first “superbug” and is linked to a high global death toll from infections resistant to antibiotics (15). The challenges posed by antimicrobial resistance (AMR) will be addressed through a variety of strategies, such as developing novel antimicrobials, strengthening the surveillance system for AMR in animal and human populations, better understanding the ecology of resistant bacteria and resistant genes, raising stakeholder awareness of the responsible use of antibiotics in animal production and clinical settings, and addressing the effects of AMR on public health and the environment (16). A thorough review of the literature incorporates previous research findings into this pilot project, particularly concerning cost estimate selection. Considering the patient’s viewpoint, the unadjusted mean additional cost of an antimicrobial-resistant infection is roughly USD 1300 compared to the susceptible group and USD 1923 compared to the uninfected cohort, according to a study conducted in Ghana. The loss of productivity is responsible for approximately one-third of the extra costs. Considering an estimated annual antimicrobial resistance (AMR) risk of 7.5% in hospitals, the projected yearly cost of AMR is approximately USD 962,000 compared to the susceptible cohort and USD 1,423,020 compared to the uninfected cohort. The extra costs resulting from AMR show a significant positive association not only with the length of stay (LOS) but also with female sex. However, in this study, the LOS due to bacteremia in the AMR cohort was 5.15 days, which was significantly greater than that in the non-infectious cohort. Among the bacterial isolates, the highest transport costs were observed for *Pseudomonas* spp. It relates to the most frequently identified pathogen in the study patients. The current study indicates that the mean length of stay (LOS) due to bacteremia is greater in cases than in non-infectious controls but lacks the additional cost due to prolonged stay in the hospital. This is supported by the report of Ireland, where hospital costs for resistant patients were around an additional €12 million in 2019. As indirect costing measures, including food costs, stay charges, and productivity loss, relate to LOS, the indirect cost burden is higher in non-infected controls than in infected cases. This reflects the gaps in the healthcare delivery system observed in HFH hospital. As empirical treatment with antibiotics is initiated without testing for culture susceptibility, LOS is increased because of failure to respond or possibly resistant drugs. In addition, if the sample is tested negative for infection, the resistance profile of the patient toward antibiotics was ignored due to a lack of resources. However, it is equally possible that non-infected patients may show resistant profiles; however, because of this gap, HFH represents no susceptible control (17). Antimicrobial resistance (AMR) has a significant impact on society, economics, and therapeutic results. Clinically, AMR causes high morbidity, mortality, and treatment failure. The majority of the research that has been published has linked antibiotic resistance to unfavorable outcomes, such as a 1.3–2 times increase in mortality, morbidity, and expense for individuals with resistant infections as opposed to susceptible ones (18). Resistance frequently causes delays in the administration of microbiologically effective therapies, as several investigations have shown, which may have unfavorable consequences. There is a significant risk of renal impairment when using colistin to treat infections caused by Acinetobacter or highly resistant *Pseudomonas*. Global projections indicate that approximately 1.2 million deaths were related to antimicrobial resistance (AMR) in 2019, and this is forecasted to increase to approximately 10 million deaths per year by 2050 if insufficient action is taken to control AMR (19). The present investigations have shown that the total cumulative cost (direct and indirect costs) for cases is greater than that for susceptible controls. This means that patients infected with resistant bacteria face a higher economic burden than individuals with susceptible bacterial infections. However, the total health costs were the highest among the three cohorts. This relates to the deficiencies of healthcare delivery systems, in which empirical treatment with antibiotics is provided without testing patients for their antimicrobial sensitivity. The current study included all blood pathogens reported by two public hospitals in Pakistan through culture sensitivity reports (CST). The number of isolated bacterial pathogens was 12, categorized into 10 codes, and the 10^th^ code was for the three rare microbes found in the study sample. The most frequent blood pathogen was found to be *Salmonella* (*n* = 19), followed by *Pseudomonas* (*n* = 17), *Klebsiella* (*n* = 14), *Escherichia* (*n* = 9), *Enterobacter* (*n* = 7), and MRSA (*n* = 5). The literature encompasses a range of elements, including study design, sample, methodology, perspective, pathogens, comparator groups, and cost estimates, which are all relevant to estimating the burden of antimicrobial resistance (AMR) and its associated costs. However, direct comparisons of economic costs across these studies prove challenging owing to variations in the approaches. The pragmatic review conducted in Ireland, encompassing 27 international studies, highlighted diverse methodologies. Some studies focus specifically on the cost of AMR per infection (20). In addition, incorrect antibiotic use in primary care is up to 55% in South Africa, 88% in Pakistan, 61% in China, and 15.4% in Canada. Mortality was measured as the proportion of patients who died during the study; of the 193 patients with bloodstream infections, the AMR cohort reported the highest number of deaths, 21 (45.65%), followed by 18 (39.13%) patients from the susceptible cohort and 7 from non-infectious controls (15.22%); thus, 12 resistant bacteria in the study resulted in 46 deaths. A comparable study that included 8 bacteria from 50 public hospitals found that over 4,700 resistant infections resulted in approximately 215 deaths and almost 5,000 DALYs. Our study did not consider the DALYs as an AMR consequence. We found that the female population was more resistant to antibiotics; this may be due to a greater number of females in the age bracket of 16–25, contradicting the evidence that AMR is more prevalent among children (21). Studies on the economic burden of AMR estimate different costs, reflecting the economic dynamics of a particular region. Similarly, variations in cost estimates have been found across countries because of differences in the pricing of healthcare services, and costs for human resources and products.

## Conclusion

AMR presents itself as a complex condition with varied impacts on individuals, encompassing diverse effects and associated expenses. This study identified more than 12 instances of antibiotic-resistant blood infections, resulting in an additional expenditure of approximately USD 33.97 (PKR 9483.2) due to prolonged length of stay (LOS) attributable to bacterial infections compared to the treatment of susceptible infections. Conversely, the indirect cost was found to be USD 55.84 (PKR 15588.3) higher in the non-infected control compared to the infected cases. Furthermore, the investigation revealed no significant disparities in direct costs, productivity loss, or mean staff time between the two hospitals. However, a notable distinction emerged in indirect costs, amounting to USD 18.10 (PKR 5053.729) across both settings. Establishing the current cost of AMR proves invaluable in guiding future investment decisions, underscoring the urgent requirement for financial support and resources to develop cost-effective solutions for addressing the challenges posed by AMR. Understanding the current cost of antimicrobial resistance (AMR) is crucial for making informed future investments, emphasizing the urgent need for funding to develop cost-effective solutions. In addition to patients, the burden of AMR on hospitals, especially in public sector settings that receive patients from multiple provinces, should be considered. Strengthening the healthcare delivery system by providing new testing tools to public hospitals is essential. Mandatory Culture Susceptibility Testing (CST) for patients requiring antibiotics and ensuring affordable diagnostic costs less than treatment costs are the recommended measures.

### Strengths and limitations

This study included both direct and indirect cost variables associated with AMR and the consequences of AMR. One limitation of the study was its restricted scope in terms of the included pathogens and cost variables considered. The length of stay was measured in terms of days. The cost per patient bed per day is missed. It would be more appropriate if the cost per patient bed per day was estimated. Similarly, instead of staff time, the cost per staff could be more appropriate to provide cost estimates. Another limitation of this study was the pricing policies of both hospitals. The rates of the two diagnostic tests (i.e., culture test and sensitivity) and antibiotics were different in both hospitals, as PIMS follows federal rates and HFH adopts Punjab rules and rates. Therefore, there is no standard cost. Both hospitals cost items differently because of local behavior, the presence or absence of automated testing, the lack of information systems, and the particular technologies adopted.

## Data Availability

The original contributions presented in the study are included in the article/supplementary material further inquiries can be directed to the corresponding author.
